# Airborne spread and infection of a novel swine-origin influenza A (H1N1) virus

**DOI:** 10.1186/1743-422X-10-204

**Published:** 2013-06-22

**Authors:** Hongna Zhang, Xin Li, Ruihua Ma, Xiaoxia Li, Yufa Zhou, Hongliang Dong, Xinxian Li, Qinglei Li, Mingliang Zhang, Zhihao Liu, Baozhi Wei, Mingchao Cui, Hao Wang, Jing Gao, Huili Yang, Peiqiang Hou, Zengmin Miao, Tongjie Chai

**Affiliations:** 1 College of Animal Science and Veterinary Medicine, Shandong Agricultural University, Daizong Street 61, Tai’an 271018, China; 2 Sino-German Cooperative Research Centre for Zoonosis of Animal Origin Shandong Province, Tai’an, Shandong, China; 3 Key Laboratory of Animal Biotechnology and Disease Control and Prevention of Shandong Province, Tai’an, Shandong, China; 4 Affiliated Hospital of the Shandong Agricultural University, Tai’an, China; 5 Taishan Medical University, Tai’an, China; 6 Centre for Disease Control, Tai’an, the People’s Republic of China, Tai’an, China; 7 The Animal Husbandry Bureau of Tai’an City, Tai’an, China

**Keywords:** S-O 2009 IV, Epidemic, Airborne transmission, Pig, Guinea pig

## Abstract

**Background:**

The novel swine-origin influenza A (H1N1) virus (S-O 2009 IV) can cause respiratory infectious diseases in humans and pigs, but there are few studies investigating the airborne spread of the virus. In January 2011, a swine-origin H1N1 epidemic emerged in eastern China that rapidly spread to neighboring farms, likely by aerosols carried by the wind.

**Methods:**

In this study, quantitative reverse transcription polymerase chain reaction (RT-PCR) was used to detect viruses in air samples from pig farms. Based on two aerosol infection models (Pig and guinea pig), we evaluated aerosol transmission and infection of the novel S-O 2009 IV isolate.

**Results:**

Three novel S-O 2009 IV were isolated from the diseased pig. The positive rate and viral loads of air samples were 26.1% and 3.14-5.72 log_10_copies/m^3^ air, respectively. In both pig and guinea pig infection models, the isolate (A/swine/Shandong/07/2011) was capable of forming aerosols and infected experimental animals at a range of 2.0-4.2 m by aerosols, but aerosol route was less efficient than direct contact.

**Conclusions:**

The results indicated that S-O 2009 IV is able to be aerosolized by infected animals and to be transmitted to susceptible animals by airborne routes.

## Introduction

In April 2009, swine-origin 2009 A (H1N1) influenza viruses (S-O 2009 IV) were found in Mexico and the United States for the first time, and quickly spread throughout the world, presenting a significant threat to public health [1,2]. S-O 2009 IV is a novel triple-reassortant influenza virus derived from porcine, human, and avian influenza viruses. Different from seasonal influenza viruses, humans lack immunity to this new virus, and thus the virus quickly caused a pandemic [2-5]. As of March 21, 2010, the World Health Organization (WHO) reported that 213 countries or regions were affected and the number of deaths was at least 16,931 people [6].

It has been determined that despite the complex causes of the novel S-O 2009 IV epidemic, airborne spread was one of the major reasons for the pandemic [7]. Aerosols are solid or liquid suspensions in the air and their particle size range is 0.001-100 μm [8]. Once formed, aerosols, including those containing viruses, can rapidly spread to a larger area with the assistance of the wind [9]. However, little is known about the transmission and infection of the novel S-O 2009 IV via aerosols, and there is still some debate about airborne infection of this virus [7,8,10,11].

In early January 2011, a local animal disease prevention and control center in Shandong, China reported that a pig farm in eastern China emerged the suspected novel S-O 2009 IV disease. More importantly, similar infections occurred successively in some downwind pig farms within a week, and workers of these pig farms developed flu-like symptoms. Isolation and identification of pathogens confirmed S-O 2009 IV infection. Thus, we speculated that airborne transmission played an important role in the spread of this epidemic. Here, we collected indoor air, pig nasopharyngeal swabs, and blood samples to analyze the positive rate of the S-O 2009 IV; and based on both pig and guinea pig aerosol infection models, airborne infection capacity of S-O 2009 IV isolate was evaluated.

## Results

### Identification of viruses

The seven viral isolates were obtained from the nasopharyngeal swab samples, including three strains of H1N1 and four strains of H3N2. Antigenicity and sequence analysis of the three strains of H1N1 viruses confirmed that the isolated strain was S-O 2009 IV. And the isolate (A/swine/Shandong/07/2011) was used in aerosol infection models to verify its airborne transmission traits.

### Results of air and serum samples

The positive rate of 157 air samples collected from the 40 pig farms was 26.1% (41/157) and the virus content range was 4.09-5.72 log_10_copies/m^3^ air; hemagglutination and hemagglutination-inhibition (HA-HI) tests showed that the positive rate of serum samples was 28.5% (57/200) and titers were 80–2560. Infection analysis of staff with flu-like symptoms from seven pig farms found that 43.5% (10/23) were seropositive rate for S-O 2009 IV infection (from CDC) (Table 1).

**Table 1:** Detection results of airborne S-O 2009 IV in 40 pig farms

**Farms**	**Air samples**	**Virus RNA copies of air samples (log**_**10**_**/m**^**3**^**)**	**Sero- Conversion**	**Farms**	**Air samples**	**Virus RNA copies of air samples (log**_**10**_**/m**^**3**^**)**	**Sero- Conversion**
Farm 1	4/6	4. 14–5.62	3/5	Farm 21	3/6	4.41-5.07	3/5
Farm 2	4/6	4.05-4.93	5/5	Farm 22	0/4	0	0/5
Farm 3	2/5	3.83/5.66	4/5	Farm 23	1/4	4.62	2/5
Farm 4	2/4	4.20/4.54	3/5	Farm 24	1/4	4.40	2/5
Farm 5	2/4	4.75/5. 19	2/5	Farm 25	1/3	4.78	1/5
Farm 6	2/4	3.74/4.64	4/5	Farm 26	1/4	3.14	2/5
Farm 7	3/4	4.20-4.63	3/5	Farm 27	0/4	0	0/5
Farm 8	3/4	4.12-5.72	4/5	Farm 28	0/3	0	0/5
Farm 9	3/4	4.55-5. 09	3/5	Farm 29	0/3	0	0/5
Farm10	0/4	0	1/5	Farm 30	0/3	0	1/5
Farm 11	0/3	0	0/5	Farm 31	0/3	0	0/5
Farm12	0/3	0	0/5	Farm 32	0/3	0	0/5
Farm13	0/4	0	0/5	Farm33	1/4	4.95	3/5
Farm14	0/4	0	0/5	Farm34	0/4	0	0/5
Farm15	1/3	5.50	0/5	Farm35	0/3	0	1/5
Farm16	0/4	0	0/5	Farm 36	0/4	0	0/5
Farm17	2/4	4.42/5.02	1/5	Farm 37	0/3	0	0/5
Farm18	1/5	5.59	1/5	Farm 38	1/4	4.65	3/5
Farm19	3/4	4. 91–5.33	4/5	Farm 39	0/4	0	0/5
Farm20	0/4	0	0/5	Farm 40	0/4	0	1/5

### Results of S-O 2009 IV aerosol infection models

All experimental pigs and guinea pigs in the challenged groups were shown to shed viruses and seroconverted to S-O 2009 IV. In the swine infection model experiments, viruses were detected in the direct contact groups from nasal secretions of three pigs during the first round of experiment and two pigs during the second round. In the aerosol infection group, two pigs were positive during the first round of experiments and one positive during the second round. Pigs with virus detected in nasal secretions were also positive by serological testing. Serum titers of the direct contact group and aerosol infection group were 320–1280 and 640–1280, respectively (Table 2).

**Table 2:** Transmission and infection of 2009 A(H1N1) IV aerosol in pig model and guinea pig model

		**Inoculated animals**		**DC animals**^**§**^		**AI animals**^**§**^	
		**Virus in nasal wash**^**#**^**(log**_**10**_**peak copies/ml)**	Sero- conversion^*^	**Virus in nasal wash**^**#**^**(log**_**10**_**peak copies/ml)**	Sero- conversion^*^	**Virus in nasal wash**^**#**^**(log**_**10**_**peak copies/ml)**	Sero- conversion^*^
Pigs	1	3/3(5.3-6.1)	3/3(640–2560)	3/3(4.9-5.6)	3/3(320–1280)	2/3(5.5-6.0)	2/3(640–1280)
	2	3/3(5.1-5.8)	3/3(640–1280)	2/3(5.1-5.6)	2/3(320–640)	1/3(4.9)	1/3(640)
Guinea Pigs	1	5/5(4.3-5.2)	5/5(640–1280)	4/5(4.2-4.8)	4/5(640–1280)	2/5(3.8-5.1)	2/5(320–640)
	2	5/5(5.4-6.2)	5/5(320–1280)	3/5(4.7-5.8)	3/5(320–1280)	3/5(4.1-6.2)	3/5(320–1280)

^§^DC direct contact; AI aerosol infection; ^#^Virus titers are expressed as mean log_10_peak copies/ml; ^*^Hemagglutination inhibition(HI)assay was performed with homologous virus and turkey red blood cells. No. of positive animals and HI range indicated.

In the guinea pig infection model experiments, viruses were detected in nasal secretions from four guinea pigs in the first round of experiments and three guinea pigs in the second round in the direct contact group. In the aerosol infection group, viruses were detected in nasal secretions from two guinea pigs for the first round of experiments and three guinea pigs for the second round. Serum titers of animals in the direct contact group and aerosol infection group were 320–1280 and 320–1280, respectively (Table 2).

**Figure 1:**
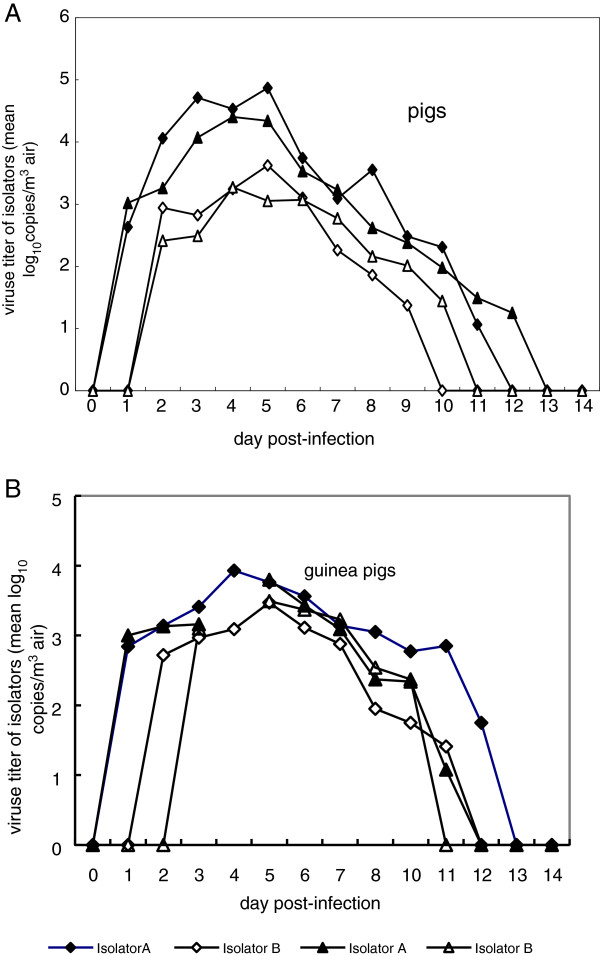
**Concentration of the SO 2009(H1N1)IV aerosol produced by pigs and guinea pigs in isolators A and B.** The diamond represents the first round of experiment; triangle represents the second. Black represents isolator A, white represents isolator B. (B) Not detected in the first round of experiment 4dpi Day 0: animals in group A w ere intranasally inoculated with 10^6^ pfu of the novel influenza A (H1N1) virus.

During the two rounds of swine infection model experiments, S-O 2009 IV aerosols were detected at the beginning of 1 day post-infection (dpi) in isolator A, peaked (4.87, 4.40 log_10_copies/m^3^ air) at 5 and 4 dpi, and was absent from 12 or 13 dpi. In isolator B, S-O 2009 IV aerosols were detected at 2 dpi, peaked (3.62, 3.27 log_10_copies/m^3^ air) at 5 and 4 dpi, and was absent after 10 or 11 dpi (Figure 1A).

For the guinea pig experimental infection model, the results of the two rounds of experiments indicated that virus shedding was detected at 1 dpi of the challenged group; S-O 2009 IV aerosols were detected at 1 and 2 dpi, peaked at 4 and 5 dpi (in isolator A and in isolator B) and disappeared at 11–13 dpi. In the two rounds of guinea pig infection model experiments, S-O 2009 IV aerosols were detected at 1 dpi in isolator A, peaked at 4 and 5 dpi (3.93, 3.80 log_10_copies/m^3^ air), and was absent from 12–13 dpi. In isolator B, SO 2009 IV aerosols were detected at post-inoculation 2 and 3 days, peaked at 5 dpi (3.47, 3.49 log_10_copies/m^3^ air), and was absent at 11 or 12 dpi (Figure 1B).

### Diameter of S-O 2009 IV aerosol particles

**Figure 2:**
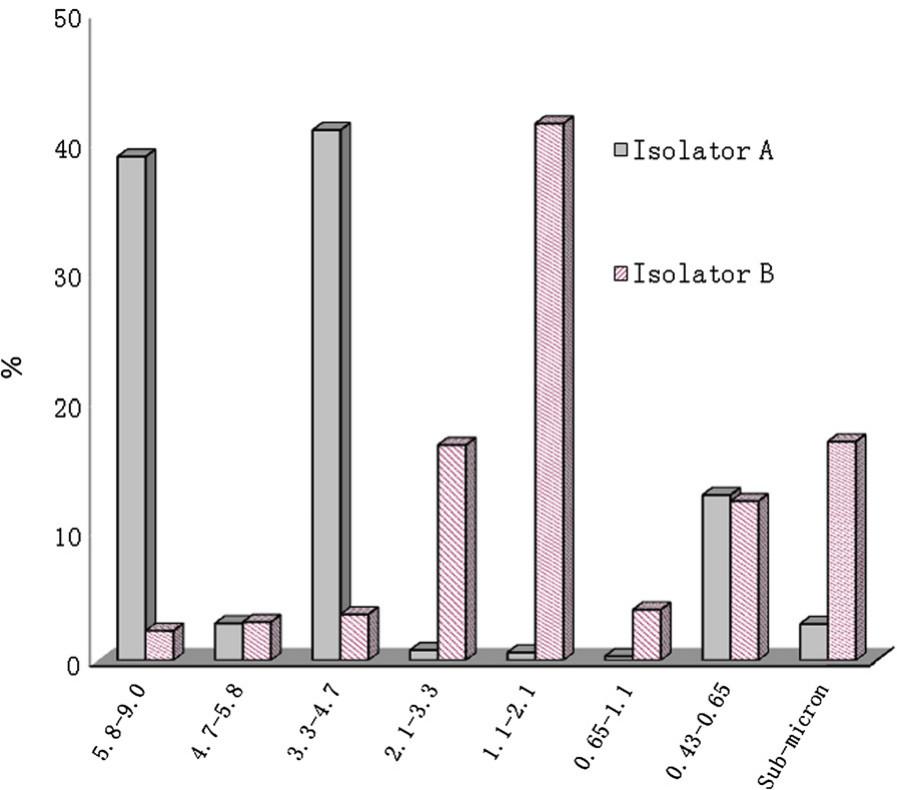
**Distribution characteristics of the novel influenza A (H1N1) virus from isolator A and B on the Andersen-8-stage impactor, Based on RT-qPCR results.** X-axis: aerodynamic diameter of airborne viral particles on Andersen-8 sampler (from left to right:1-8 stage); Y-axis: the distribution percentage of viral particles on every stage of Andersen-8 sampler.

In isolator A, the distribution ratio of viral aerosol particle sizes was highest for a particle diameter of 3.3-4.7 μm (40.95%), and lowest for 0.65-1.1 μm (0.30%). In isolator B, the distribution ratio of viral aerosol particle sizes was highest for particle diameters of 1.1-2.1 μm (41.45%), and lowest for 5.8-9.0 μm (2.28%) (Figure 2).

## Discussion

Since early January 2011, the S-O 2009 IV epidemic was first identified in two pig farms of eastern China that quickly spread to nearly 20 farms within a week. A geographical analysis of these pig farms showed that these farms were distributed in a close ellipsoid zone with a central axis in line with prevailing northwesterly winds. A collection of 157 air samples from 40 pig farms revealed that 26.1% contained virus with almost 50% of pig farms in the area affected. This transmission was presumed to be via downwind dissemination of S-O 2009 IV aerosols. It appears that the virus was spread by airborne transmission and our findings in experimental models support this.

**Figure 3:**
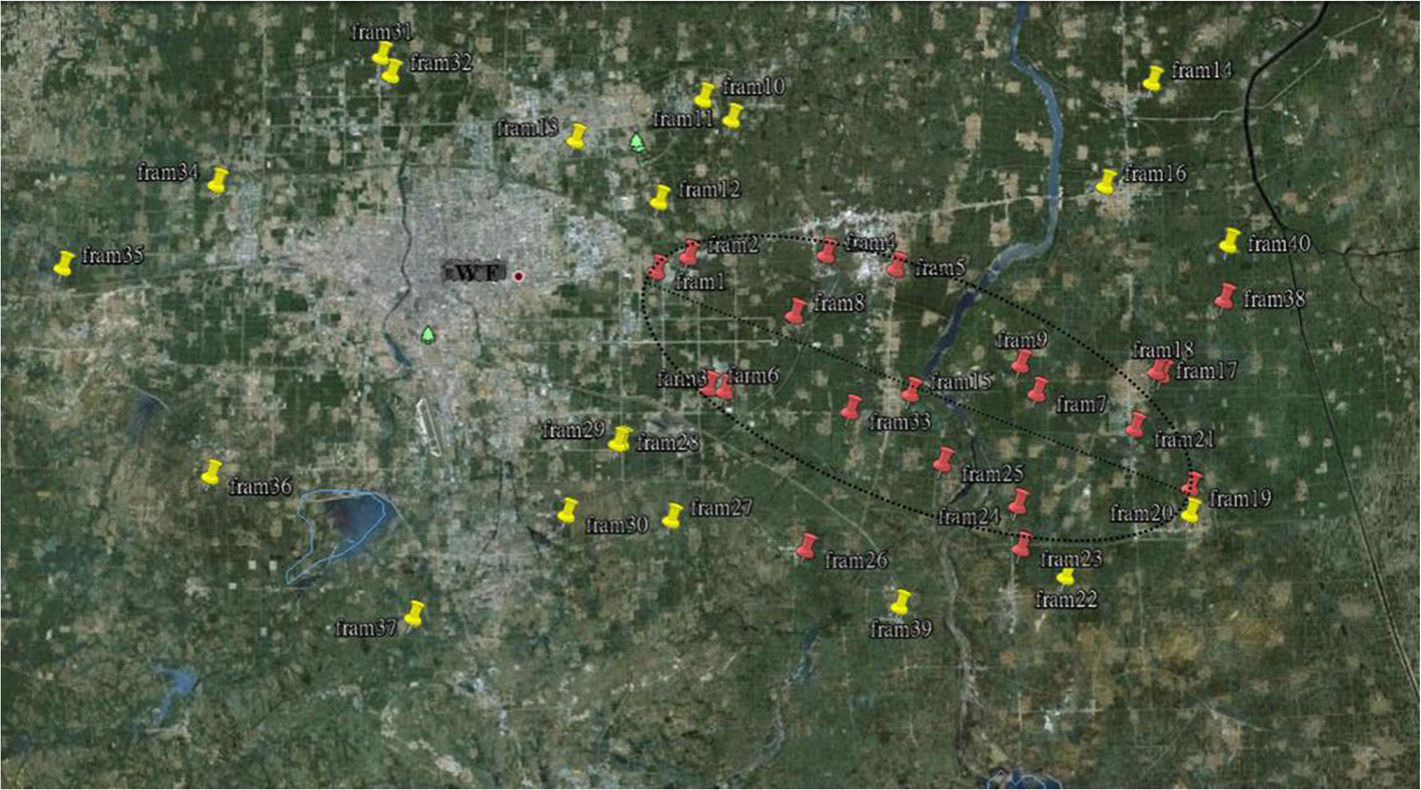
The map is the part of East China area, red points represent the positive pig farms, and yellow ones represent the negative pig farms.

During the experiments, pigs and guinea pigs were housed in positive- and negative-pressure isolators, effectively avoiding the contamination with exogenous microbes. Aerosol transmission experiments were conducted so that the air in isolator B came exclusively from isolator A, which were connected using a 2 m closed tube. Therefore, any infection in isolator B would be determined as aerosols (Figure 3).

During the experimental infections in pigs, S-O 2009 IV was detected at 1–2 dpi in isolators A and B, and the maximum amount of virus detected in A and B were and 4.40-4.87 log_10_copies/m3 air and 3.27-3.62 log10copies/m3 air, at 4 and 5 dpi, respectively. However, during the two rounds of experiments, the amounts of virus detected were consistently lower in B than in A and virus was not detectable at 12–13 dpi in A and at 10–11 dpi in B. The duration of detectable S-O 2009 IV aerosols in isolator B was shorter and the amounts of virus were lower than those in isolator A, which was likely related with the deposition, survival time, and removal of aerosol particles.

**Figure 4:**
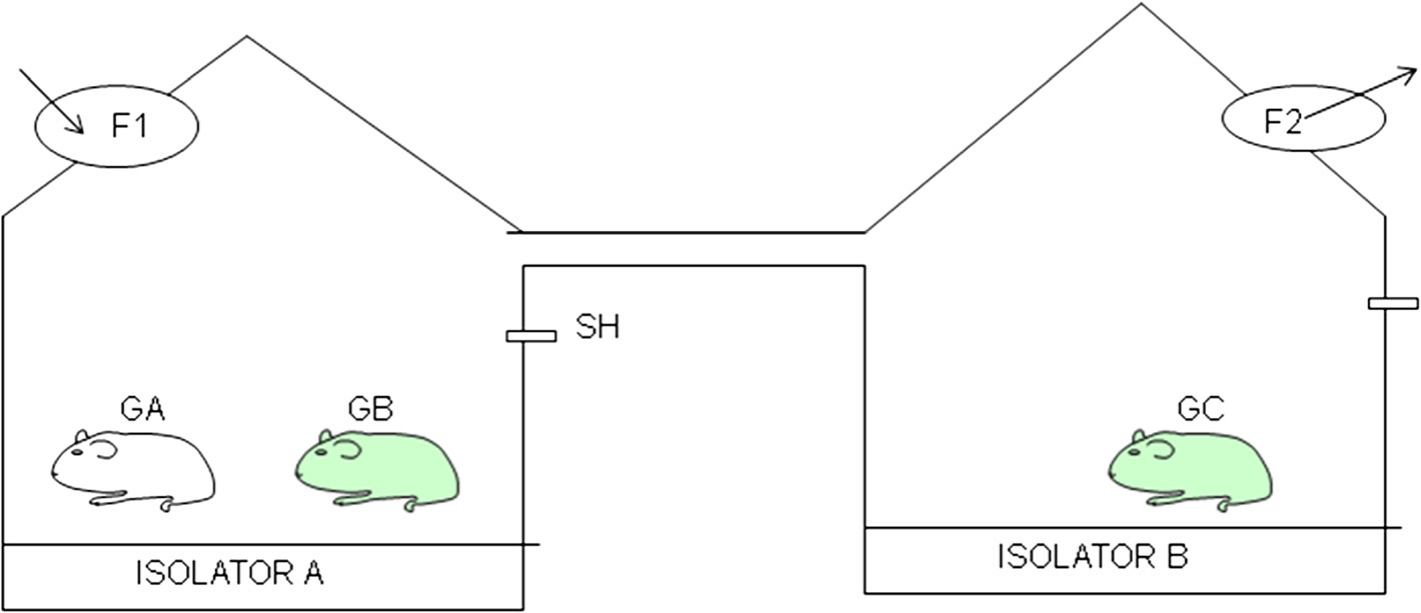
**Arrangement of the isolators applicable to pigs and guinea pigs.** Animals in inoculation group (GA) and direct contact group (GB) were housed in the isolator A, and animals in aerosol infection group (GC) were housed in the isolator B. The two isolators were connected with a tube(2 m) which allowed the air flowed from A to B. SH is a sampling hole from which air samples were collected. F1 is a positive pressure fan and F2 is a negative fan. The air flowing through the two fans was filtered to remove microorganisms.

There is a 2m pipe between the two isolators (upper distal end, 5.3m from the challenged group; Figure 4); therefore, infection in isolator B occurred not via droplet but by aerosolized virus particles. Meanwhile the certain wind velocity in the pipe can well simulate natural wind between different pig farms, even between different pig houses in the same farm. Additionally, aerosol infection group and direct contact group were both serologically positive and nasal secretions had evidence of virus shedding. Nonetheless, the number of infections in aerosol infection group was smaller than in direct contact group, there was no difference in the extent of virus shedding and serum titers of infected pigs. These data demonstrated that infection with these viral strains can induce airborne infection.

Our data indicated that infected pigs located in upwind farms can generate viruses in nasal and respiratory secretions and spread via the wind to downwind farms. Thus, this type of transmission is dependent on weather, where changes in wind force, wind direction, ultraviolet light, temperature or humidity are all likely to affect transmission.

Since the respiratory tract of guinea pigs is very similar to that of humans, guinea pigs were regarded as an appropriate mammalian model for use in this study [12]. In the two repeated trials on guinea pigs, the rates of virus detection and seroconversion to the S-O 2009 IV in aerosol infection group were 2/5 and 3/5 (Table 2), indicating that aerosol infection group was infected by viral aerosols in isolator A and the virus had the capability of airborne transmission among mammals. Therefore, it can be inferred that S-O 2009 IV is likely to be a health risk for farm staff and may be an important point for the prevention and control of new zoonoses, especially swine influenza viruses. It has been reported that the S-O 2009 IV is still persistent among pigs in China and thus remains a major threat to human health [13,14].

At 4 and 5 dpi, the virus content in air samples reached its peak in the isolator along with the amount of virus in nasal secretions and the number of pigs infected in different groups. These data indicated that the amount of virus contained in aerosols is related to the virus shedding capacity and number of infected animals. Until 11–13 dpi, no airborne S-O 2009 IV was detected in the two isolators. Correspondingly, animals in all the experimental groups no longer shed virus. These results suggest that the viruses produced in nasal secretions by animals formed aerosols.

Although the detectable time and concentrations of airborne virus in the two animal experiments were different, the viral aerosol concentration increased or decreased coincidentally. In addition, the viral aerosol concentration was affected by the ventilation rate and the number of infected animals.

The aerodynamic diameter ranges of aerosols in isolator A were determined to be 3.3-4.7 μm and 5.8-9.0 μm; however, in isolator B particle size was typically 1.1-2.1 μm. The loss of large particle droplets was mainly due to sedimentation, and the loss of aerosol particles <5μm was mainly due to the inactivation of virus particles and ventilation [15], indicating that smaller particles gathered in isolator B under the effect of ventilation. In the two isolators, particles were typically ≤ 4.7 μm, showing that a higher proportion of viral aerosols can enter the lower respiratory tract via the nasal cavity, whereas particles ≤1 μm can enter bronchioles and alveoli, and deposit in the alveoli, increasing the risk of serious infection. Gustin et al. [16] indicated that when discharged by breathing or sneezing in ferrets, influenza virus particles were typically ≤4.7 μm. During the flu season, the less than 4 μm (aerodynamic diameter) influenza viral aerosol particles accounted for 53% of the airborne influenza viral count in hospital environments [17-19], consistent with the experimental results of this study. In conclusion, animals infected with S-O 2009 IV can form aerosols that can lead to airborne transmission.

## Materials and methods

### Outbreak of the epidemic and virus isolation and identification

The swine flu epidemic occurred in eastern China (119° E, 36.30° N), a warm temperate semi-humid monsoon climate zone, with cold, dry winters dominated by northwesterly winds [20]. In early January 2011, a suspected new swine flu epidemic suddenly emerged in a few pig farms of this region and within a week similar epidemics emerged successively in some pig farms in the downwind direction of this pig farm (Figure 3). A total of 120 nasopharyngeal swabs taken from infected pigs were collected for virus isolation and identification, as previously described by Cong et al. [21]. Samples were processed in a BSL −2+ laboratory.

### Collection and process of air, nasopharyngeal swab and blood samples

A total of 157 air samples were collected using AGI-30 (All Glass Impinger) placed in the middle of the pig farm, at 1.5 m from the ground, using 20 mL phosphate buffered saline (PBS) sampling medium, with a flow velocity of 12.5 L/min for 40 min [22,23]. Virus was detected in samples using the RT-qPCR methods [24]. Serum samples from pigs (n=200) and pig farm staff (n=23) displaying flu-like symptoms were collected and processed by the local centers for disease control (CDC) [25].

### Establishment of S-O 2009 IV aerosols transmission and infection models

Weaned piglets (8–10 kg) and guinea pigs (250–300 g) seronegative for S-O 2009 IV were purchased from the Shandong Taibang Biological Product Co., Ltd. And used for experimental infections. All prevailing local, national and international regulations and conventions, and normal scientific ethical practices have been respected in this study. Two positive- and negative-pressure isolators A and B (Model C.C.JH-1; Tianjin Jinhang Purified Air Conditioning Engineering Company, China) were connected using a closed tube 2 m in length and 8 cm in diameter, to adjust air flow from A to B, which not only prevented direct contact of animals in the aerosol infection group with those in the inoculation group, but also prevented the propagation of droplets (Figure 4). Three pigs or five guinea pigs were intranasally inoculated with 10^6^ plaque-forming units (PFU) of viruses were placed in isolator A, and were designated inoculation group. After 24 h, another three pigs or five guinea pigs were placed into isolator A, and were designated direct contact group. Three pigs or five guinea pigs were placed into isolator B, and were designated aerosol infection group [26,27]. The isolator temperature was maintained at 20±1 °C, 30±4 % relative humidity (RH), and 0.05-0.2 m/s wind velocity [28]. Pig and guinea pig experiments were conducted independently and repeated. “The Society for Animal Health Feedings & Animal Welfare of Shandong Province approved the study of “Airbone spread and infection of a novel swine-origin influenza A(H1N1) virus” and agreed to exam the serum samples from 200 pigs and 23 pig farm staff for the experiments of that.

### Collection of aerosols, nasal washes, and blood in infection experiments

After sequencing and analysis of eight fragments from several virulent strains, three strains were identified as the S-O 2009 IV and one of them was used for the aerosol dissemination experiment. Virus shedding and serum antibodies of nasal secretions in experimental animals were monitored and detected to verify if the experimental animals were infected. Nasal secretions from each animal were collected every other day after virus inoculation and after RNA extraction, virus nucleic acids were detected by RT-qPCR [29]. At 7 and 14 dpi, blood from each experiment animal was collected to test for specific antibodies [30].

After inoculation, the AGI-30 air collection device was used to collect air samples in isolators A and B on each day [26] and the virus content in samples was detected using RT-qPCR. To determine the proportion and distribution of aerosol particles of different sizes, at 4 dpi of the second round of the guinea pig model experiments, the Andersen-8-level collector [18] was used to collect aerosolized viral particles in isolators A and B, using a sterile1% gelatin glycerol (1% gelatin PBS and glycerol 1:1 mixed) as the medium, at 28.3 L/min flow velocity for 40 min, and the virus content of every level was detected by RT-qPCR.

## Competing interests

The authors declare they have no competing financial interests.

## Authors’ contributions

HZ and XL are contributed equally to this work. RM, XL, YZ, JG, HY, ZM to participate in the writing of this paper. HD, XL, QL, MZ, ZL, BW, MC, HW and PH participate in the operation of the experiment. All authors read and approved the final manuscript.

## References

[B1] Zepeda-Lopez HM Perea-Araujol L Miliar-Garca1 A Dominguez-Lopez A Xoconostle-Cazarez B Lara-Padilla E Inside the outbreak of the 2009 influenza A(H1N1)v virus in Mexico PLoS One 2010 5 e13256 10.1371/journal.pone.0013256 20949040 PMC2951908

[B2] Novel swine-origin influenza A(H1N1) virus investigation team Emergence of a novel swine-origin influenza A(H1N1) virus in humans N Engl J Med 2009 360 2605 2615 19423869 10.1056/NEJMoa0903810

[B3] Seema J Laurie K Anna MB Ann MS Stephen RB Janice L Hospitalized patients with 2009 H1N1 influenza in the United States, April–June 2009 N Engl J Med 2009 361 1935 1944 10.1056/NEJMoa0906695 19815859

[B4] Jonathan RK Swine influenza J Clin Pathol 2009 62 577 578 10.1136/jcp.2009.067710 19433408

[B5] Vincent JM de Emmie W van den Judith MA van den B Sander H Eefje JAS Theo MB Pathogenesis and transmission of swine-origin 2009 A(H1N1) influenza virus in ferrets Science 2009 325 481 483 19574348 10.1126/science.1177127 PMC4814155

[B6] Wang HJ Update of influenza A [H1N1] Jilin Medicine 2011 32 133 135

[B7] Maines TR Jayaraman A Belser JA Wadford DA Pappas C Zeng H Transmission and pathogenesis of swine-origin 2009 A(H1N1) influenza viruses in ferrets and mice Science 2009 325 5939 484 487 19574347 10.1126/science.1177238 PMC2953552

[B8] Tellier R Aerosol transmission of influenza A virus: a review of new studies J R Soc Interface 2009 6 S783 S790 10.1098/rsif.2009.0302.focus 19773292 PMC2843947

[B9] Xihua Y The infection and control of microoganism aerosol-concurrently treats of prevent in the SARS virus Contamination Control & Air-Conditioning Technology 2003 4 25 2923819106

[B10] Wit E Vincent JM Riel D Walter EPB Rimmelzwaan GF Kuiken T Molecular determinants of adaptation of highly pathogenic avian influenza H7N7 viruses to efficient replication in the human host J Virol 2010 84 1597 1606 10.1128/JVI.01783-09 19939933 PMC2812334

[B11] Piso RJ Albrecht Y Handschin P Bassett S Low transmission rate of 2009 H1N1 influenza duringa long-distance bus trip Infection 2011 39 149 153 10.1007/s15010-011-0084-x 21340580 PMC7099280

[B12] Sun YP Bi YH Pu J Hu YX Wang JJ Gao HJ Guinea pig model for evaluating the potential public health risk of swine and avian influenza viruses PLoS One 2010 5 e15537 10.1371/journal.pone.0015537 21124850 PMC2990763

[B13] Howden KJ Brockhoff EJ Caya FD Mclecod LJ Lavoie M Ing JD An investigation into human pandemic influenza virus (H1N1)2009 on an Albeta swine farm Can Vet J 2009 50 p1153 p1161 PMC2764467 20119537

[B14] Zhao G Pan JJ Gu XB Lu XL Li QH Hu J Isolation and phylogenetic analysis of avian-origin European H1N1 swine influenza viruses in Jiangsu China Virus Genes 2011 44 11

[B15] Yang W Marr LC Dynamics of airborne influenza A viruses indoors and dependence on humidity PLoS One 2011 6 e21481 10.1371/journal.pone.0021481 21731764 PMC3123350

[B16] Gustin KM Belser JA Wadford DA Pearce MB Katz JM Tumpey TM Influenza virus aerosol exposure and analytical system for ferrets PNAS 2011 108 8432 8437 10.1073/pnas.1100768108 21536880 PMC3100970

[B17] Blachere FM Lindsley WG Pearce TA Anderson SE Fisher M Khakoo R Measurement of airborne influenza virus in a hospital emergency department Clin Infect Dis 2009 48 438 440 10.1086/596478 19133798

[B18] Andersen AA New sampler for the collection, sizing, and enumeration of viable airborne particles J Bacterial 1958 76 471 484 10.1128/jb.76.5.471-484.1958 PMC290224 13598704

[B19] Chai TJ Ma RH Mueller W Wang HR Study on microbiological floras in air of supplying materials workshop in a biological refuse treatment Plant J Environ Health 2000 6 358 363

[B20] Zhang J Yang WZ Guo YJ Xu H Zhang Y Li Z Epidemiologic characteristics of influenza in China from 2001 to 2003 Chin J Epidemiol 2004 25 461 465 15231116

[B21] Cong YL Pu J Liu QF Wang S Zhang GZ Zhang XL Antigenic and genetic characterization of H9N2 swine influenza viruses in China J Gen Virol 2007 88 2035 2041 10.1099/vir.0.82783-0 17554038

[B22] Brachman PS Ehrlich R Eichenwald HF Gabelli VJ Kethley TW Madin SH Standard sampler for assay of airborne microorganisms Science 1964 144 1295

[B23] Chapin A Rule A Gibson K Buckley T Schwab K Airborne multidrug-resistant bacteria isolated from a concentrated swine feeding operation Environ Health Perspect 2005 113 137 142 15687049 10.1289/ehp.7473 PMC1277855

[B24] Poon LL Chan KH Smith GJ Leung CSW Guan Y Yuen KY Molecular detection of a novel human influenza (H1N1) of pandemic potentia by conventional and real-time quantitative RT-PCR assays Clin Chem 2009 55 1555 1558 10.1373/clinchem.2009.130229 19439731 PMC7108475

[B25] Luo XS Li KM Huang JD Lin JR Li JX Seroprevalence of swine influenza H1N1 virus C J Vet Med 2011 47 39 40

[B26] Yao ML Zhang XX Gao J Chai TJ Miao ZM Ma W The occurrence and transmission characteristic of airborne H9N2 avian influenza virus Berl Munch Tieraerztl Wochschr 2011 124 10 15 21462864

[B27] Li XX Chai TJ Wang ZL Song CP Cao HJ Liu JB Occurrence and transmission of Newcastle disease virus aerosol originating from infected chickens under experimental conditions Vet Microbiol 2009 136 226 232 10.1016/j.vetmic.2008.11.002 19091492

[B28] Lowen AC Steel J Mubareka S Palese P High temperature (30°C) blocks aerosol but not contact transmission of influenza virus J Virol 2008 82 5650 5652 10.1128/JVI.00325-08 18367530 PMC2395183

[B29] Lowen AC Mubareka S Tumpey TM García-Sastre A Palese P The guinea pig as a transmission model for human influenza viruses PNAS 2006 103 26 9988 9992 10.1073/pnas.0604157103 16785447 PMC1502566

[B30] Yao HC Experiment guidance for veterinarian microbiology 2007 25 Beijing: China Agriculture Press 105 107

